# Incidence and Risk Factors for Incisional Hernia Following Ileostomy Takedown: A Retrospective Cohort Study

**DOI:** 10.3390/jcm14103597

**Published:** 2025-05-21

**Authors:** Tamás Talpai, Flaviu-Ionuţ Faur, Cătălin-Alexandru Pîrvu, Daniela Marinescu, Cristi Tarta, Dragos Nicolae Margaritescu, Stelian Pantea, Cristian Nica, Rãzvan-Sorin Albu, Tudor-Alexandru Popoiu, Razvan Lazea, Larisa Balanoiu, Valeriu Șurlin

**Affiliations:** 1 Doctoral School, Department of Surgery, University of Medicine and Pharmacy of Craiova, 200349 Craiova, Romania; talpai.tamas@umft.ro (T.T.); vsurlin@gmail.com (V.Ș.); 2 X Department of General Surgery, “Victor Babes” University of Medicine and Pharmacy Timisoara, 300041 Timisoara, Romania; flaviu.faur@umft.ro (F.-I.F.); tarta.cristi@umft.ro (C.T.); pantea.stelian@umft.ro (S.P.); nica.cristian@umft.ro (C.N.); popoiu.tudor@umft.ro (T.-A.P.); razvanwpa@gmail.com (R.L.); balanoiu.larisa@gmail.com (L.B.); 3 IIIrd Surgery Clinic of “Pius Brinzeu”, County Emergency Clinical Hospital Timisoara, 300723 Timisoara, Romania; 4 IInd Surgery Clinic, Timisoara Emergency County Hospital, 300723 Timisoara, Romania; albu.razvan96@yahoo.com; 5 Department of Surgery I, University of Medicine and Pharmacy of Craiova, 200349 Craiova, Romania; dragos.margaritescu@umfcv.ro; 6 Doctoral School, “Victor Babes” University of Medicine and Pharmacy Timisoara, Eftimie Murgu Square 2, 300041 Timisoara, Romania

**Keywords:** incisional hernia, ileostomy, ileostomy-site incisional hernia, colorectal cancer, closure

## Abstract

**Background**: Incisional hernias are a frequent complication following ileostomy closure, with rates reaching 24%. Protective ileostomies are commonly performed in colorectal surgery, but their closure presents a significant risk for abdominal wall defects. Identifying risk factors for incisional hernias at the ileostomy site is crucial for improving patient outcomes. **Methods**: This retrospective study analyzed data from 95 patients who underwent loop ileostomy closure at two Romanian hospitals between 2018 and 2023. Patient demographics, surgical details, and follow-up data were reviewed. Incisional hernias were diagnosed through clinical examination or radiological imaging. Statistical analyses, including univariate and multivariate regression, were performed to identify independent risk factors. **Results**: The incidence of incisional hernias at the ileostomy site was 13.7% (13/95). Univariate analysis identified BMI (HR 30.08; *p* = 0.007), previous hernia (HR 7.99; *p* = 0.059), radiotherapy (HR 299.15; *p* = 0.029), and chemotherapy (HR 0.004; *p* = 0.026) as significant factors. Multivariate analysis confirmed BMI > 30 kg/m^2^ (HR 12.27; *p* = 0.002) and prior hernia (HR 8.14; *p* = 0.007) as independent risk factors. **Conclusions**: Obesity and previous hernias significantly increase the risk of incisional hernias following ileostomy closure. Radiological follow-up enhances early detection, and further studies should explore the benefits of prophylactic mesh reinforcement. Optimizing patient selection and surgical technique may reduce postoperative hernia rates, improving long-term outcomes.

## 1. Introduction

Incisional hernias are a common complication following abdominal surgery, with an estimated incidence of up to 20% after laparotomy [1]. These hernias can result in significant morbidity, including pain, bowel obstruction, and the need for surgical repair. Protective ileostomies, commonly created to divert fecal flow and facilitate the healing of an intestinal anastomosis, are linked to a notably elevated incidence of incisional hernia formation upon closure [2]. The rationale for their creation may differ, ranging from low rectal tumors to acute diverticulitis or inflammatory bowel disease [3]. Numerous risk factors have been reviewed in the literature, encompassing, patient demographics, comorbidities, and technical aspects of the surgical procedure [4,5,6]. Furthermore, the time frame until ileostomy closure may affect local tissue integrity and the likelihood of hernia development. The closure duration typically ranges from 3 to 6 months; however, various factors may prolong this process, including adjuvant chemotherapy, preexisting medical comorbidities, or surgeon discretion, sometimes resulting in the conversion of a temporary ileostomy into a permanent stoma [7]. Understanding the incidence and risk factors related to incisional hernias following ileostomy closure is essential for enhancing patient outcomes and reducing the impact of this complication.

Despite loop ileostomy reversal being seen as a relatively straightforward procedure, incisional hernia rates have been documented to reach as high as 24% post-closure [6]. This risk appears to be higher than that seen with other abdominal operations, which may be related to the altered anatomy and tissue quality at the ileostomy site. Patient-related risk factors that have been associated with increased incisional hernia rates include obesity, diabetes, smoking, and poor nutritional status [8]. Various technical aspects have been considered, including the type of suture employed for skin closure (linear versus purse-string), the method of fascial closure, and the reinforcement of the abdominal wall using mesh [9,10].

This study seeks to assess the incidence and risk variables associated with incisional hernia development following ileostomy closure by identifying and quantifying pertinent risk factors in a 5-year cohort, utilizing clinical and radiological data from 95 patients.

## 2. Materials and Methods

This study retrospectively reviewed data from patients who underwent loop ileostomy closure at three surgical departments in Romania: Craiova Emergency Clinical County Hospital’s 1st surgical clinic and Timișoara County Emergency Clinical Hospital’s 2nd and 3rd clinics, spanning a five-year period from 2018 to 2023.

The Institutional Review Board of the participating hospital granted ethical approval for this study, ensuring compliance with the ethical standards outlined in the 1964 Helsinki Declaration and its subsequent amendments related to human research ethics. The study’s protocol was reviewed and approved, having each approval registered through their Institutional Regional Board. Local ethics committee approval for this study was obtained (No. 262/27 November 2023).

### Data Collection

Inclusion criteria included patients aged 18 years and older who underwent planned loop ileostomy closure. Participants in the study had a history of either benign or malignant colorectal disease. Index surgery refers to the procedure involving colorectal resection, applicable to both benign and malignant conditions. Patients who met the inclusion criterion of having undergone a protective loop ileostomy closure were identified by reviewing electronic medical records. Post-ileostomy closure follow-up involved outpatient clinical visits and periodic CT evaluations, particularly for patients with malignant disease, conducted at 6 months, 1 year, and 2 years following primary resection of the tumor.

Demographic factors, including age and gender, alongside clinical factors such as body mass index, preoperative hemoglobin, length of stay, ASA score, smoking status, presence of diabetes mellitus, chronic respiratory disease, and history of abdominal wall hernias, as well as neoadjuvant radiotherapy or chemotherapy, were extracted. Intraoperative data were recorded, encompassing the type of initial colorectal surgery (elective or emergency), technical approach (laparoscopy vs. laparotomy), time to ileostomy closure, Clavien–Dindo score, and surgical technique for skin closure.

Surgical interventions primarily involved procedures on the left colon and rectum for malignancies, along with a limited number of miscellaneous indications, including trauma, acute diverticulitis, and fulminant inflammatory bowel disease.

The primary outcome assessed was the incidence of incisional hernia at the ileostomy site post-closure, defined by the European Hernia Society as any defect in the abdominal wall, with or without a bulge, detectable through examination or imaging studies within the postoperative scar [11]. Defects were identified during routine clinical examinations conducted by surgeons registered in each institution’s electronic registry; through high-resolution computed tomography follow-ups performed by radiologists, which included descriptions of the defects, their contents, and their sizes in the reports; or when surgical repair was proposed for these defects, with data extracted from the same electronic registries.

The surgical technique for the primary intervention was conducted in accordance with oncological principles in cases of malignancy, leading up to the creation of a loop ileostomy at the end of the procedure. A minor fascial incision was created in the right lower quadrant to exteriorize the ileal loop, which was secured in two layers: one to the fascia and ileal serosa and another between the mucosa and the skin, facilitating the eversion of the future stoma to prevent local complications. During the initial postoperative period, antibiotics were administered only if an infectious source was found and complications were reported according to the Clavien–Dindo classification, with appropriate treatment provided [11].

The timing of ileostomy closure was established by the surgeon, factoring in the likelihood of anastomotic leak, the need for adjuvant chemotherapy, and the patient’s comorbidities. Preoperative verification of the colorectal anastomosis was carried out using flexible endoscopy, contrast-enhanced enema CT, or radiography. An elliptical incision was made around the stoma, following the dissection of the loop from the skin and fascia and the resection of the previous intestinal defect, which was followed by a side-to-side intestinal anastomosis. Primary fascial closure was successfully accomplished in all cases using interrupted stitches, without specification of the suture material, following either a purse-string or linear primary closure of the overlying skin.

Surgical repair was proposed for an incisional hernia diagnosed at the ileostomy closure site. An open retromuscular repair was utilized in each instance, accessing the defect via a lateral incision for isolated defects, or through the midline when an incisional hernia was concurrently identified at this location.

Continuous data were described using means ± standard deviations (SDs), ranges (minimum–maximum), and medians (interquartile ranges). Categorical data were described using frequency and percentage. Continuous quantitative variables were analyzed via univariate analyses, and multivariate analyses were performed when a significant relationship was found between univariate ones. Data analysis was performed using GraphPad PRISM (ver. 10.1.1, 2023). The statistical significance threshold was established at the 0.05 level (2-sided).

## 3. Results

**Figure 1:**
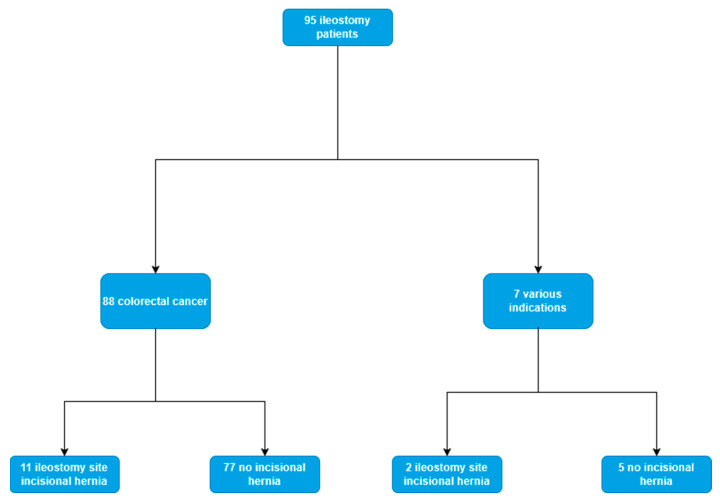
Flowchart with main patient outcomes.

Between 2018 and 2023, following the exclusion criteria, a total of 95 patients were included in the study. Median age at enrollment was 62 years, with a slight prevalence of males over females (66.3% male vs. 33.7% female). Most of the patients included in the study received a loop ileostomy for fecal diversion following colorectal excision for malignancy (88/95); however, seven patients included had other indications for its formation (trauma, inflammatory bowel disease, anastomotic leak after right hemicolectomy). The median follow-up from ileostomy closure was 22.47 months (IQR 9.5–39.9 months) (Figure 1). Baseline characteristics of all patients are shown in Table 1. A laparoscopic approach was employed in 21 patients (22.1%) and open in 74 (77.9%). Taking into consideration that the majority of patients underwent surgery for malignancy, the ASA score distribution was as follows: two were grade 2 (3.2%), eighty-eight were grade 3 (92.6%), and four met the criteria for grade 4 (4.2%). In the immediate postoperative period, the rate of complications measured using the Clavien–Dindo classification was as follows: grade I 23/95 (24.21%), grade II 36/95 (37.9%), grade IIIa 11/95 (11.6%). Applying the same system at the time of ileostomy closure, we obtain the following results: grade 0, 48/95 (50.5%), grade I, 26/95 (27.4%), grade II, 18/95 (18.9%), grade IIIa, two (2.1%), and grade IIIb, one (1.1%).

**Figure 2:**
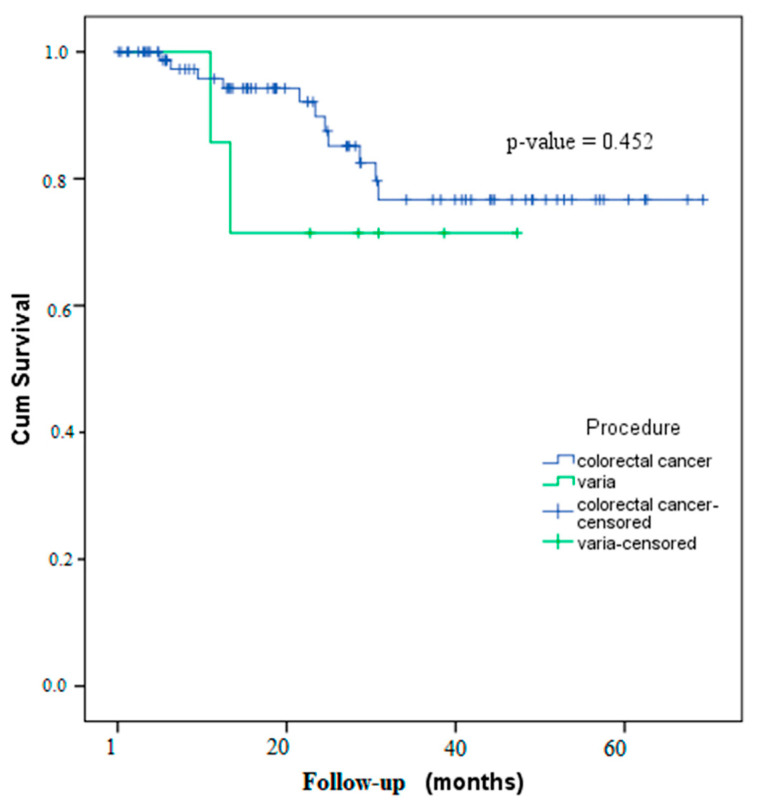
Kaplan–Meier curve for incisional hernia-free survival.

The mean duration from the initial surgery to ileostomy takedown was 4.99 ± 3.6 months (range: 0–16.1 months). A longer duration was observed in patients without colorectal cancer (6.2 ± 4.4 months; range: 1.6–14.1 months), although this difference did not achieve statistical significance (*p* = 0.459) (Figure 2).

### Risk Factors for Ileostomy Incisional Hernia

The incidence of incisional hernias at the previous ileostomy site was 13.7% (*n* = 13/95). The mean age at diagnosis was 61.23 years (range: 48–79), with no significant difference observed in comparison to individuals who did not experience this complication. Among these cases, eight were identified through oncological follow-up via CT imaging, while five were detected during routine clinical examinations. Furthermore, among the thirteen patients who developed an incisional hernia at the ileostomy site, eleven had an ileostomy created for colorectal malignancy (11/88, 12.5%), while two were for other, previously mentioned indications (2/7, 28.6%).

In our univariate regression analysis (Table 2), BMI above 30 kg/m^2^ (HR 30.08; 95%CI 2.53–357.6; *p* = 0.007), previous hernia (HR 7.99; 95%CI 0.92–69,13; *p* = 0.059), radiotherapy (HR 299.15; 95%CI 1.8–49650; *p* = 0.029), and chemotherapy (HR 0.004; 95%CI 0–0.524; *p* = 0.026) were found to be correlated with increased rate of ileostomy-site incisional hernia. In our multivariate regression analysis (Table 2), only two variables proved to be independent risk factors for ileostomy: BMI (HR 12.27; 95%CI 2.46–61.15; *p* = 0.002) and previous hernia (HR 8.14; 95%CI 1.76–37.75; *p* = 0.007).

Although the number of subjects included in the study with other indications for ileostomy formation and closure besides colorectal cancer had a reduced number (*n* = 7), statistically significant results were obtained with regards to patient BMI (25.8 ± 3.1 vs. 28.89 ± 3.59, *p* = 0.027) and emergency surgery (71.4% vs. 10.2%, *p* = 0.001).

## 4. Discussion

Loop ileostomies are adjunct procedures typically performed at the conclusion of a surgical intervention, before the closure of the abdominal wall. These are primarily utilized for the protection of anastomoses in colorectal surgeries, the management of inflammatory bowel diseases, and the treatment of complications in colorectal cancer patients [12]. However, their application may extend to other contexts, including trauma surgery following rectal perforations [13]. Despite concerns about their effectiveness in reducing colorectal anastomosis leak rates in recent years, they have demonstrated a capacity to mitigate the clinical consequences of leaks by sustaining the stoma until both clinical and radiological resolution of the bowel wall defect occur [14]. Given the large variety of scenarios for which they are being performed, and also the necessity to maintain them, the time to their takedown may vary considerably. The mean duration for ileostomy closure varies, ranging from 8 to 13 days to over 12 weeks, with certain cases encountering considerable delays attributed to external factors [15]. Early closure, occurring within 4–6 weeks, is typically regarded as safe and feasible for selected patients. In contrast, conventional practices frequently postpone closure to 2–3 months to ensure sufficient healing and minimize complications [7,16,17]. In our study, the mean duration until takedown, expressed in months, was 4.99 ± 3.6.

Stoma-site incisional hernias remain a major source of morbidity and increase the patients’ dependency on healthcare providers [8]. Barranquero et al. showed that in a single-institution retrospective cohort study, which included 133 patients with various indications for their loop ileostomy, diagnosed either via clinical exam or radiological methods, the rate of stoma-site hernia was 11.6% (15/133) [18]. In comparison, Kaneko et al. conducted a retrospective study involving 134 oncological patients, diagnosed solely through routine computed tomography scans, and reported an incidence of 23.9% (32/134) [6]. The reported rates vary between 6.1% and 23.9%, with elevated rates noted in studies featuring extended follow-up periods and stricter diagnostic criteria [6,8]. The incidence of stoma-site incisional hernias in our cohort of 107 patients was 13.7% (*n* = 13/95), aligning with intervals reported by previous authors.

Regarding follow-up, it has been recognized that longer follow-up times only increase their incidence, with no specific cut-off point at which the risk becomes minimal, and although the majority of them occur within the first year, incisional hernias have been reported as far as 43 months after reversal [19]. In the same study conducted by Kaneko et al., with a median follow-up of 47 months, 23.9% of patients developed incisional hernias, with the median detection time being 8 months [6]. A study by Kelly-Schuette et al. reported a median detection time of 16.4 months within a follow-up period of 49.5 months, and another study by Brook et al. reported a median follow-up time of 20.5 months, with the majority of incisional hernias occurring at a median of 8 months post-reversal [4,19]. The mean follow-up duration in our study was 25.29 months, while the average time until defect diagnosis was 18.61 months. The discrepancies in detection times reported by different authors can be attributed to the diagnostic methods utilized. Notably, ultrasound and/or computed tomography may enhance diagnostic accuracy, particularly in patients with significant adipose tissue [20].

Although clinical examination remains the most common method for diagnosing abdominal wall defects, it has been shown to be inferior to CT for detecting incisional hernias, with 23% missed by the former and 31% missed in obese patients [21]. Abdominal ultrasound and computed tomography can be utilized for the diagnosis of incisional hernias [20]. Ultrasound is non-invasive, cost-effective, and involves no radiation, rendering it appropriate for numerous patients. However, inter-observer variability may reduce its sensitivity and specificity [22]. Computed tomography, despite its higher cost and radiation exposure, offers enhanced anatomical insights and may influence the surgical strategy depending on the size and characteristics of the hernia, making it a superior choice for complex abdominal wall repairs [23]. One study found that Dynamic Abdominal Sonography for Hernia (DASH) had a sensitivity of 98% and specificity of 88% [20]. Moreso, CT scans are highly reliable for diagnosing incisional hernias, with studies demonstrating a sensitivity of 79% and specificity of 94% for hernia detection, and given the necessity for colorectal cancer patients to have routine follow-ups, their diagnosis may be facilitated through this procedure [21]. In our study, among the thirteen patients diagnosed with an ileostomy-site incisional hernia, five were found via clinical exam usually undertaken by their surgeon (38.46%) and eight through computed tomography and/or ultrasound routinely performed in the context of oncological follow-up (61.54%).

The incidence of incisional hernia at the ileostomy site may vary based on the underlying indication for the procedure. The group mostly included colorectal cancer patients (eighty-eight out of ninety-five, or 92.63%) compared to those with non-cancer conditions (seven out of ninety-five, or 7.37%), which made it hard to find a statistical difference in the results. However, Schuette et al. observed that loop ileostomies created for acute diverticulitis, which were later reversed, constituted only 19% of their cases but accounted for 62% of the identified stoma-site incisional hernias (18/29) [4].

Recent investigations have identified several risk factors for incisional hernias at ileostomy sites, many of which overlap with those associated with other site-specific defects, including midline, subcostal, and non-midline hernias [24]. The primary risk factors for developing an incisional hernia at the ileostomy site include age and gender, obesity, hypertension, history of previous abdominal wall hernias and significant postoperative complications [2]. Specific surgical technical factors have been identified, including the methods used for fascial closure and the type of skin closure employed [10]. A considerable number of patient-related factors can be identified but cannot be adequately treated or adjusted for during the perioperative period, primarily due to the inability to cancel or postpone surgeries for malignant conditions [25].

Obesity is consistently identified as a significant risk factor for the development of incisional hernias after ileostomy closure. Higher BMI increases the likelihood of hernia formation across multiple studies [26,27,28]. This may be due to the increased tangential strain on the abdominal wall in obese patients, which is due to their wider ventral wall radius [29]. Furthermore, the substantial subcutaneous fat layer in these patients may hinder the visibility of the fascia during closure, potentially elevating the risk of a technique breach. Our multivariate regression analysis indicated that a BMI exceeding 30 kg/m^2^ is associated with a heightened risk of ileostomy-site hernias (HR = 2.46–61.15; *p* = 0.002).

Previous hernias, characterized as any primary, non-site-specific abdominal wall defect (inguinal, midline, lumbar, etc.) that was either repaired or diagnosed during the initial surgery, have been hypothesized to be a risk factor for subsequent hernia formation. This hypothesis suggests that altered collagen synthesis may lead to reduced resistance of the abdominal wall [30]. Other authors have also investigated it as an independent risk factor. Fazekas et al. reported a hazard ratio of 3.59 (*p* = 0.0087) in their cohort of 121 exclusively colorectal cancer patients, and Kaneko et al. found that patients with concomitant midline incisional defects showed an increased risk for ileostomy-site incisional hernias (OR = 5.63, *p* = 0.0003) [2,6]. Consequently, in our study, patients with synchronous midline abdominal defects did not exhibit an elevated risk for the primary outcome (HR = 2.94, 0.38–22.79, *p* = 0.303), but patients with previous hernias had a statistically significant increased risk for ileostomy-site incisional hernias. (HR = 8.14, 1.76–37.75, *p* = 0.007).

In the end, while this technique was not utilized in our study group, prophylactic mesh reinforcement following ileostomy closure has been extensively examined in prior literature, emphasizing its advantages and drawbacks and advocating for a patient-specific strategy [9,18,31]. Research indicates that the use of prophylactic mesh during ileostomy closure markedly decreases the incidence of incisional hernias. One study indicated that 6.4% of patients with mesh developed hernias, in contrast to 36.1% in the non-mesh group. Additionally, Olona et al. reported no hernias in the mesh group, while the control group exhibited an 11% incidence [9,31]. Mesh type and characteristics varied across studies, but overall prosthetic reinforcement during closure did not significantly increase the risk of surgical site infections [32,33].

## 5. Conclusions

Ileostomy-site incisional hernias remain a constant issue during follow-up for patients, with a reported incidence of 13.7% in our cohort. Established risk factors for their development include a BMI exceeding 30 kg/m^2^, prior hernia surgery, and a history of radio- and chemotherapy, which have been identified as reliable predictors. High-risk patients may derive the greatest advantage from prophylactic mesh reinforcement to decrease incidence rates. Additional research is necessary to enhance our predictive capacity and to identify specific factors related to surgical techniques, including the types of fascial and skin closure.

## Figures and Tables

**Table 1:** Baseline demographics for patients undergoing an ileostomy.

	*n* = 95	Indication		
		Colorectal Cancer (*n* = 88)	Other Indications (*n* = 7)	*p*-Value
Age, years				0.231
Mean ± SD	61.95 ± 9.79	62.24 ± 9.95	58.3 ± 7	
Median (IQR)	62 (56–68)	62 (56–68)	59 (52–63)	
Range	36–84	36–84	48–69	
Gender, male *n* (%)	63 (66.3%)	59 (67%)	4 (57.1%)	0.684
Weight (kg)				0.01
Mean ± standard deviation	85.27 ± 10.43	86.05 ± 10.03	75.6 ± 11.3	
Median (IQR)	87 (80–91)	87 (80–91.75)	78 (74–83)	
Range	52–110	60–110	52–87	
Body mass index (kg/m^2^)				0.027
Mean ± SD	28.66 ± 3.63	28.89 ± 3.59	25.8 ± 3.1	
Median (IQR)	28.4 (26.5–30.9)	28.7 (26.9–31)	26.3 (24.2–27.9)	
Minimum–maximum	19.8–37.9	20.7–37.9	19.8–29	
Hypertension, yes	80 (84.2%)	75 (85.2%)	5 (71.4%)	0.305
Diabetes mellitus, yes	20 (21.1%)	19 (21.6%)	1 (14.3%)	1.00
Chronic obstructive pulmonary disease, yes	16 (16.8%)	15 (17%)	1 (14.3%)	1.00
Smoking, yes	61 (64.2%)	57 (64.8%)	4 (57.1%)	0.698
Previous hernia, yes	19 (20%)	18 (20.5%)	1 (14.3%)	1.00
Adjuvant radiotherapy, yes	64 (67.4%)	62 (70.5%)	2 (28.6%)	0.036
Adjuvant chemotherapy, yes	60 (63.2%)	59 (67%)	1 (14.3%)	0.009
Type of surgical approach				0.648
Laparoscopy	21 (22.1%)	19 (21.6%)	2 (28.6%)	
Open	74 (77.9%)	69 (78.4%)	5 (71.4%)	
Type				0.001
Urgency	14 (14.7%)	9 (10.2%	5 (71.4%)	
Elective	81 (85.3%)	79 (89.8%)	2 (28.6%)	
ASA score				0.74
2	3 (3.2%)	3 (3.4%)	-	
3	88 (92.6%)	81 (92%)	7 (100%)	
4	4 (4.2%)	4 (4.5%)	-	
Hb preoperatory				0.598
Mean ± SD	12.3 ± 1.8	12.3 ± 1.8	12.8 ± 1.6	
Median (IQR)	12.4 (11.2–13.5)	12.4 (11.2–13.4)	12.6 (10.8–14.4)	
Range	7.7–15.9	7.7–15.9	10.8–14.6	
Clavien–Dindo				0.265
Grade I	48 (50.5%)	46 (52.3%)	2 (28.6%)	
Grade II	36 (37.9%)	33 (37.5%)	3 (42.9%)	
Grade IIIa	11 (11.6%)	9 (10.2%)	2 (28.6%)	
Clavien–Dindo at takedown				0.648
0	48 (50.5%)	46 (52.3%)	2 (28.6%)	
Grade I	26 (27.4%)	23 (26.1%)	3 (42.9%)	
Grade II	18 (18.9%)	16 (18.2%)	2 (28.6%)	
Grade IIIa	2 (2.1%)	2 (2.3%)	-	
Grade IIIb	1 (1.1%)	1 (1.1%)	-	
Skin closure				0.415
Secondary	62 (65.3%)	56 (63.6%)	6 (85.7%)	
Primary	33 (34.7%)	32 (36.4%)	1 (14.3%)	
Days of hospitalization				0.490
Mean ± SD	12.42 ± 8.7	12.4 ± 8.9	12.3 ± 4.6	
Median (IQR)	9 (8–14)	9 (8–14)	11 (8–16)	
Range	6–66	6–66	8–20	
Parastomal hernia	10 (10.5%)	9 (10.2%)	1 (14.3%)	0.553
Synchronous midline incisional hernia	8 (8.4%)	7 (8%)	1 (14.3%)	0.471
Ileostomy-site incisional hernia	13 (13.7%)	11 (12.5%)	2 (28.6%)	0.244
Time to ileostomy takedown				0.459
Mean ± SD	4.99 ± 3.6	4.9 ± 3.5	6.2 ± 4.4	
Median (IQR)	3.91 (2.1–7.6)	3.9 (2.04–7.6)	6.96 (2.6–8.2)	
Range	0–16.1	0–16.1	1.6–14.1	
Follow-up				0.503
Mean ± SD	25.3 ± 18.3	25.1 ± 18.7	27.5 ± 13.1	
Median (IQR)	22.47 (9.5–39.9)	20.6 (8.6–40.5)	28.5 (13.3–38.6)	
Range	0.1–69.29	0.1–69.29	10.97–47.3	

**Table 2:** Univariate and multivariate analysis of baseline predictors of incisional hernias at ileostomy site.

Characteristics	Univariate Analysis		Multivariate Analysis	
	HR (95% CI)	*p*-Value	HR (95% CI)	*p*-Value
Age	0.82 (0.09–7.88)	0.866		
Type of skin closure	0.03 (0.001–1.65)	0.086		
Body mass index (BMI)	30.08 (2.53–357.6)	0.007	12.27 (2.46–61.15)	0.002
Follow-up time	0.95 (0.88–1.02)	0.153		
Smoking status	1.94 (0.34–11.16)	0.46		
Procedure	22.48 (0.4–1278.5)	0.131		
History of previous hernia	7.99 (0.92–69.13)	0.059	8.14 (1.76–37.75)	0.007
History of radiotherapy	299.15 (1.8–49650)	0.029	7.29 (0.37–144.77)	0.193
History of chemotherapy	0.004 (0–0.524)	0.026	0.13 (0.01–2.25)	0.162
Synchron. midline hernia	2.94 (0.38–22.79)	0.303		
Clavien–Dindo complication	0.49 (0.05–4.7)	0.534		
Type of skin closure	1.48 (0.07–33.32)	0.806		
Open approach	0.83 (0.09–7.56)	0.866		

## Data Availability

Data are available on request from the authors.
